# Multiple Myeloma and Alkaline Phosphatase

**DOI:** 10.4274/tjh.2014.0495

**Published:** 2015-05-08

**Authors:** İrfan Yavaşoğlu, Gürhan Kadıköylü, Zahit Bolaman

**Affiliations:** 1 Adnan Menderes University Faculty of Medicine, Division of Hematology, Aydın, Turkey

**Keywords:** Alkaline phosphatase, Myeloma, Vitamin D deficiency

## TO THE EDITOR

The article entitled “Bone-Specific Alkaline Phosphatase Levels among Patients with Multiple Myeloma Receiving Various Therapy Options”, written by Çetin et al. [1] and published in a recent issue of your journal, was quite interesting. Here we would like to emphasize some relevant points.

This study was not homogeneous. It is important that the initial value of total alkaline phosphatase (ALP) be measured. In addition, in that study, had serum intact parathyroid hormone level, vitamin D level, or bone turnover markers been available, they would have provided further biochemical correlates of skeletal metabolism.

Bone-specific ALP may increase in Paget’s disease, osteosarcoma, bone metastases of prostatic cancer (high/very high ALP values), other bone metastases, fractured bones, multiple myeloma (only when associated with fractures), osteomalacia, rickets, vitamin D deficiency (moderate rise), malignant tumors (ALP originating from tumors), renal disease (secondary hyperparathyroidism), and primary hypothyroidism [2,3].

Vitamin D deficiency is extremely common in multiple myeloma, with 40% of patients having vitamin D levels in the deficient range of levels less than 36 nmol/L, and it represents a surrogate for clinical multiple myeloma disease status [4].

Vitamin D deficiency can cause osteomalacia and those patients can develop generalized musculoskeletal pain, proximal muscle weakness, and increased risk of falls. This may not be clinically detectable but is nonetheless present and often unrecognized [5].

**Conflict of Interest Statement**

The authors of this paper have no conflict of interest, including specific financial interests, relationships, and/or affiliations relevant to the subject matter or materials included in this manuscript.
