# Supplementary material for: Multiple Myeloma and Alkaline Phosphatase
Source: Turk J Haematol. 2015 May 8;32(2):189–90. doi: 10.4274/tjh.2014.0495 (PMC4706085; doi:10.4274/tjh.2014.0495)
Supplement: Related texts

# Reply: Authors' Response

**Authors:** Güven Çetin

## REPLY

At the beginning of the study we had planned to use some other parameters with a standard multiple myeloma work-up. We consulted with biostatisticians. As we compared a wide variety of patient groups in this analysis, according to the biostatisticians adding new parameters to our data would complicate the study. Therefore, we had to exclude some parameters.

We thank the reviewers for their valuable comments. We agree to plan further studies with new therapy options in small patient groups adding serum intact parathyroid hormone level, vitamin D level, and bone turnover.
